# Analysis of MicroRNA Regulation and Gene Expression Variability in Single Cell Data

**DOI:** 10.3390/jpm12101750

**Published:** 2022-10-21

**Authors:** Wendao Liu, Noam Shomron

**Affiliations:** 1 Faculty of Medicine, Tel Aviv University, Tel Aviv 69978, Israel; 2 Edmond J. Safra Center for Bioinformatics, Tel Aviv University, Tel Aviv 6329302, Israel

**Keywords:** microRNA, gene expression, single cell, data analysis

## Abstract

MicroRNAs (miRNAs) regulate gene expression by binding to mRNAs, and thus reduce target gene expression levels and expression variability, also known as ‘noise’. Single-cell RNA sequencing (scRNA-seq) technology has been used to study miRNA and mRNA expression in single cells. To evaluate scRNA-seq as a tool for investigating miRNA regulation, we analyzed datasets with both mRNA and miRNA expression in single-cell format. We found that miRNAs slightly reduce the expression noise of target genes; however, this effect is easily masked by strong technical noise from scRNA-seq. We suggest improvements aimed at reducing technical noise, which can be implemented in experimental design and computational analysis prior to running scRNA-seq. Our study provides useful guidelines for experiments that evaluate the effect of miRNAs on mRNA expression from scRNA-seq.

## 1. Introduction

miRNAs are small non-coding RNA molecules that regulate gene expression in metazoan organisms. miRNAs function post-transcriptionally by regulating target genes through facilitated mRNA degradation or translational repression; this potentially reduces mRNA and protein levels [1,2,3]. In addition to affecting the level of gene expression, miRNAs reduce gene expression variability, or ‘noise’, particularly of less expressed genes [4,5]. This latter effect has been hypothesized to reduce stochasticity in gene expression and to confer robustness to genetic pathways [6].

Single-cell RNA sequencing (scRNA-seq) is a rapidly developing technology that enables direct profiling of gene expression in single-cell resolution. Quantification of cell-to-cell variation using scRNA-seq provides deep insights into expression level heterogeneity and stochastic gene expression [7]. A derivative of scRNA-seq, single-cell small RNA sequencing, reveals the expression pattern of the small-sized fraction of RNAs, including (among others) miRNAs, tRNAs, and small nucleolar RNAs [8]. The majority of studies that investigated the miRNA-mRNA regulatory interplay integrated miRNA-mRNA data from ‘bulk’ sequencing experiments (as opposed to single cell), and focused on the expected anti-correlative expression levels between the miRNAs and their target genes [9,10]. Those studies were limited in their ability to measure the effect of miRNAs on the variability of mRNA expression. However, advancements in single-cell mRNA and miRNA sequencing technologies now enable exploring the interplay between miRNA levels and noisy gene expression [11].

Drawbacks of scRNA-seq data include the occurrence of stochastic noise, which is due to the sampling method, the small amount of starting material, and sequencing inefficiency [12]. Approaches that have been proposed to resolve these technicalities include the use of unique molecular identifier (UMI) [13] and external RNA spike-ins [14]. However, technical noise is greater in scRNA-seq than in bulk RNA-seq (non-single cell); hence, accurate quantification and decomposition of technical and biological noise in scRNA-seq remain challenging. Our study evaluated the effect of miRNA on mRNA expression from single-cell RNA sequencing. We showed that the conclusions that can be derived from this analysis are limited, and proposed a few ideas for improvement.

## 2. Materials and Methods

### 2.1. MRNA and MiRNA Profiling Datasets

In GSE81287 [8], single-cell mRNA sequencing was conducted according to the Smart-seq2 protocol [15], and single-cell miRNA expression was measured with small RNA sequencing. Smart-seq2 sequencing data of 21 human naive embryonic stem cells (ESCs) and 21 primed ESCs were available. The data of other types of cells were not included in the analysis. Samples with library size < 0.1 million were discarded in the analysis; thus, 16 naive ESCs and 15 primed ESCs were included.

The raw read count and RPKM matrices of mRNAs were downloaded from GEO (https://ftp.ncbi.nlm.nih.gov/geo/series/GSE81nnn/GSE81287/suppl/GSE81287_smartseq2_refseq_rpkms.txt.gz accessed on 1 December 2019). Genes expressed in more than five naive ESCs or five primed ESCs were retained in the analysis.

The molecule count matrix of miRNAs was downloaded from GEO (https://ftp.ncbi.nlm.nih.gov/geo/series/GSE81nnn/GSE81287/suppl/GSE81287_allcells_mirna_postqc.txt.gz accessed on 1 December 2019). Samples sequenced with 51 bp reads (41 bp RNA fragments) were used to calculate miRNA expression. To maintain consistency, we normalized read count similarly as with the other dataset used in our analysis, GSE114071. The raw read count of each miRNA was first divided by the sum count of all miRNAs. Normalized data lower than 10e−4 were further set to this minimum level. Then, all the data were log2-transformed for downstream analysis. Differentially expressed miRNAs were provided in the supplementary file of Omid R Faridani’s paper [8] (https://static-content.springer.com/esm/art%3A10.1038%2Fnbt.3701/MediaObjects/41587_2016_BFnbt3701_MOESM4_ESM.pdf accessed on 10 April 2022), determined using SCDE [16].

In GSE114071, the authors managed to sequence mRNA and miRNA in the same K562 single cells, using a half-cell genomics approach [17]. In short, the single cells were manually picked and lysed; the lysate of each single cell was then evenly split into two half-cell fractions. One fraction was subjected to mRNA sequencing using the Smart-seq protocol and the other was subjected to miRNA sequencing.

The normalized miRNA expression matrix was downloaded from GEO (https://ftp.ncbi.nlm.nih.gov/geo/series/GSE114nnn/GSE114071/suppl/GSE114071_NW_scsmRNA_K562_norm_log2.gct.gz accessed on 1 December 2019). The normalized expression values represented the fraction that each miRNA constituted the total miRNA content. The authors did not provide the read count for gene expression matrices, and only the RPKM matrix is available on GEO. DCA requires a read count matrix as input; therefore, we downloaded the raw mRNA sequencing data from SRA (https://www.ncbi.nlm.nih.gov/Traces/study/?acc=PRJNA464059 accessed on 1 December 2019) and quantified the gene expression ourselves. Downloaded sra files were decompressed using fastq-dump v2.8.0 and then mapped to hg38 transcriptome using STAR v2.5.3a [18]. Aligned reads were quantified using RSEM v1.3.1 [19] to obtain expected read count and RPKM values. The gene annotation used for expression quantification was the NCBI RefSeq gtf file, retrieved by Table Browser at UCSC Genome Browser (accessed on 7 December 2019). Consistent with the read count matrix, the RPKM matrix output by RSEM was used, rather than the RPKM matrix provided by the authors. Genes expressed in more than five single cells were retained in the analysis.

### 2.2. Noise Estimation and miRNA Target Prediction

**Figure 1:**
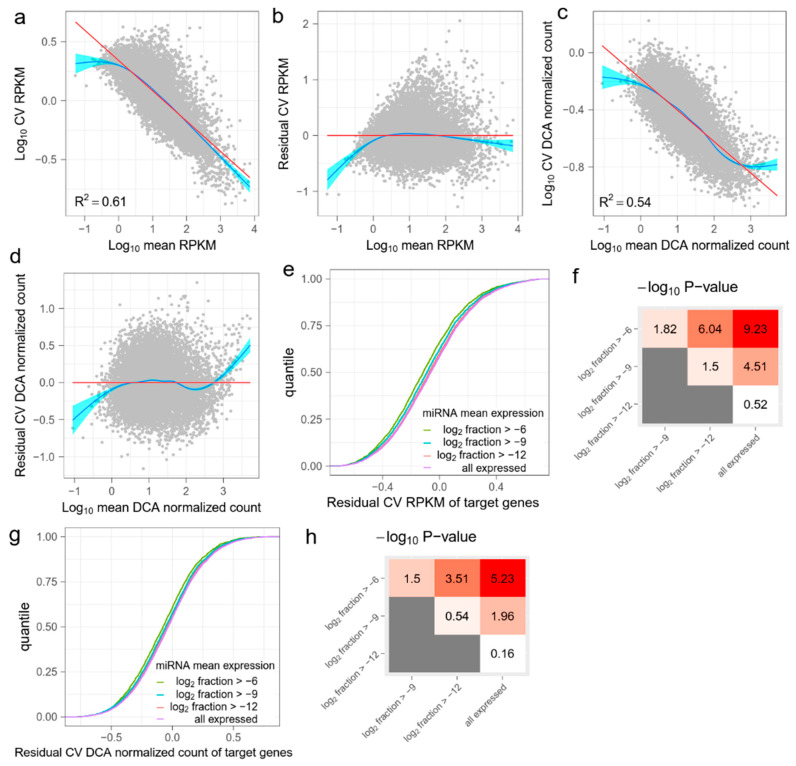
The effect of miRNA on target mRNAs in GSE81287 primed ESCs. (**a**) The linear relation between the mean and the coefficient of variation (CV) of RPKM values of genes. The red line is fitted by ordinary least squares regression and the blue line is fitted by the smoothing method, Generalized Additive Models. (**b**) Residual CV (RCV) RPKM is defined as log10 (CV) RPKM, after regressing out the mean RPKM. Overall, RCV RPKM measures the relative noise of mRNAs compared with the mean noise of mRNAs with the same expression level. (**c**) Raw read counts are denoised by DCA and then normalized. The relation between the mean and the CV of DCA normalized counts is also linear. (**d**) RCV is also calculated for DCA normalized data. (**e**) Cumulative distribution functions (CDFs) of RCV RPKM of target mRNAs. Target genes are grouped by miRNA mean expression levels. As the miRNA mean expression level increases, the RCV RPKM of the target genes significantly decreases. (**f**) Kolmogorov–Smirnov (KS) test −log10 (*p*) values between CDFs in (**e**). Red indicates higher statistical significance and white indicates lower statistical significance. (**g**) CDFs of RCV DCA normalized count of target genes. Target genes are grouped by miRNA mean expression levels. (**h**) KS-test −log10 (*p*) values between CDFs in (**g**). Red indicates higher statistical significance and white indicates lower statistical significance.

As the coefficient of variation (CV) and the mean of normalized counts are linearly related in the logarithmic scale (Figure 1), we fitted a regression line with RPKM of all the genes. Residual CV (RCV) was defined as the residual in the regression model for each gene, namely, the difference between log10 (CV) and log10 (fitted mean CV), as predicted by the regression model. This regressed out the effect of the mean expression level on noise.

Deep Count Autoencoder (DCA) was designed by Eraslan et al. to denoise scRNA-seq datasets [20]. In the DCA algorithm, a zero-inflated negative bimodal (ZINB) distribution is used to model sparse and overdispersed single-cell count data. An autoencoder framework is used to estimate the parameters of ZINB distribution, and the mean parameter of the distribution is output as the denoised count. During model training, the count data are first compressed through a bottleneck layer, where only the essential latent features are captured by the autoencoder. Therefore, random noises are largely removed in the data reconstruction step. According to Eraslan et al.’s results, DCA managed to remove technical noise and improve several downstream analyses, such as clustering, time course modeling, and differential expression analysis. We ran DCA v0.2.2 with default settings on read count matrices of GSE81287 and GSE114071 separately. Output ‘mean.tsv’ files were used as denoised count matrices. We further normalized denoised counts by the effective gene length and library size, to create DCA normalized counts. CV and RCV were also calculated for DCA normalized counts, as for RPKM values. In addition to DCA, we used another consensus clustering-based dropout imputation method for denoising, namely, ccImpute [21]. The output denoised counts were normalized using the same approach as with the DCA output.

The target genes of each miRNA were predicted using TargetScanHuman v7.2 (http://www.targetscan.org/cgi-bin/targetscan/data_download.vert72.cgi accessed on 10 July 2018) [22]. TargetScan prediction results only include conserved targets of conserved miRNA families. These targets are the default prediction results obtained from browsing the name of one miRNA on the TargetScan website. In the analysis of the effect of miRNA on target genes, the target genes of multiple miRNAs were defined as the union of target genes of each miRNA. We also included two other miRNA target prediction databases for validation, namely, miRDB 6.0 (http://www.mirdb.org/download.html accessed on 10 July 2018) [23] and miRTarBase 7.0 (http://mirtarbase.mbc.nctu.edu.tw/php/download.php accessed on 10 July 2018, latest version at https://mirtarbase.cuhk.edu.cn/~miRTarBase/miRTarBase_2022/php/download.php accessed on 17 October 2022) [24]. miRDB predicts miRNA targets using machine learning methods, while miRTarBase curates experimentally validated miRNA-target interactions.

### 2.3. Statistical Analysis

Statistical significance (*p* value) was determined by the Kolmogorov–Smirnov (KS) test for different distributions of expression noises, and the Student’s *t*-test (two-tailed) for values in naive ESCs, primed ESCs, and K562 cells. All statistical analyses were performed using R v4.0.2.

## 3. Results

To examine the effect of miRNA on gene expression, we accessed two datasets that contained both single-cell mRNA profiling and single-cell miRNA profiling. In dataset GSE81287, generated by Omid R Faridani et al., the authors applied single-cell small RNA sequencing to naive and primed human ESCs (hESCs) and to cancer cells [8]. Single-cell mRNA sequencing was also applied to naive and primed hESCs for reference. Therefore, we selected hESCs for our analysis. In dataset GSE114071, generated by Nayi Wang et al., miRNAs and mRNAs in 20 K562 cells were co-sequenced using a half-cell genomics approach [17]. These two datasets employed a read-based Smart-seq2 protocol for mRNA sequencing [15]. Therefore, in both datasets, gene expression was quantified by normalized read count such as RPKM (reads per kilobase of transcript per million reads mapped). To the best of our knowledge, no study has co-sequenced mRNA and miRNA in single cells using a UMI-based mRNA sequencing protocol.

**Figure A1:**
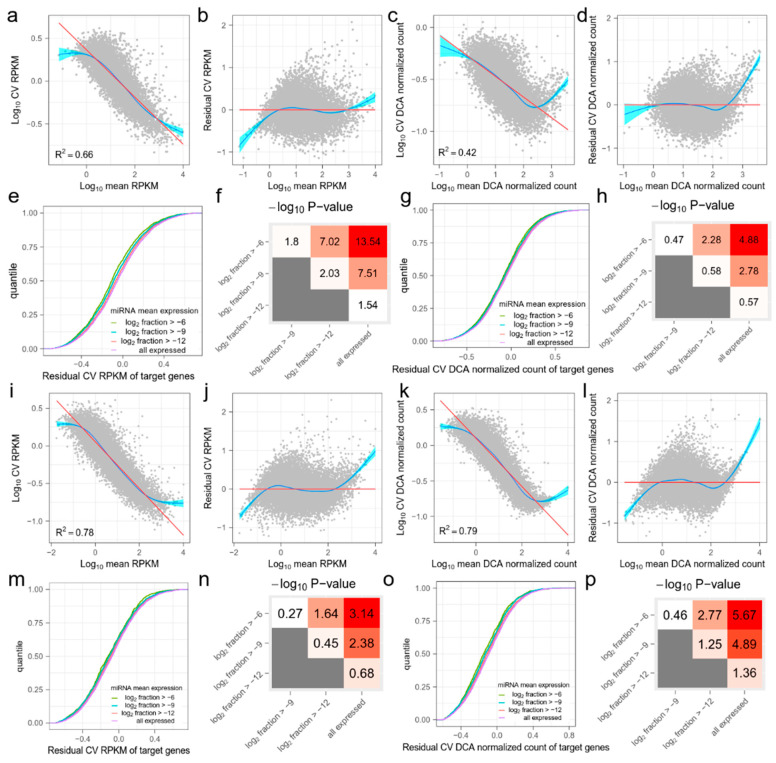
The effect of miRNA on the target mRNAs in (**a**–**h**) GSE81287 naive ESCs and (**i**–**p**) GSE114071 K562 cells. The organization of this figure is the same as for Figure 1. (**a**,**i**) The mean and coefficient of variation (CV) of RPKM values of mRNAs. The red line is fitted by ordinary least squares regression and the blue line is fitted by the smoothing method, Generalized Additive Models. (**b**,**j**) Mean and residual CV (RCV) RPKM values of mRNAs. (**c**,**k**) Mean and CV DCA normalized counts of mRNAs. (**d**,**l**) Mean and CV DCA normalized counts of mRNAs. (**e**,**m**) Cumulative distribution functions (CDFs) of RCV RPKM of target genes. Target genes are grouped by miRNA mean expression levels. As the miRNA mean expression level increases, the RCV RPKM of their target genes significantly decreases. (**f**,**n**) Kolmogorov–Smirnov (KS)-test −log10 (*p*) values between CDFs in (**e**,**m**). Red indicates higher statistical significance and white indicates lower statistical significance. (**g**,**o**) CDFs of RCV DCA normalized count of target genes. Target genes are grouped by miRNA mean expression levels. (**h**,**p**) KS-test −log10 (*p*) values between CDFs in (**g**,**o**). Red indicates higher statistical significance and white indicates lower statistical significance.

Previous studies have described a general tendency of decreasing the expression noise of mRNAs, when the mean expression level increases [25,26]. We observed that the mean and CV of normalized counts such as RPKM always showed a linear relationship in the logarithmic scale (Figure 1a and Figure A1a,i). To account for the effect of the mean expression level, we calculated RCV by subtracting the confounding mean (Figure 1b and Figure A1b,j). Accordingly, RCV measures the relative noise of one mRNA compared with the mean noise of another mRNA with the same expression level. We assume that RCVs of mRNAs should be reduced if the mRNAs are targeted by highly expressed miRNAs.

The low capture rate of single-cell sequencing technology results in drop-out events, which are failures to detect expressed genes. These events produce “false” zero count observation and may lead to biased estimations of mRNA expression. A number of methods have been proposed to denoise single-cell gene expression data and reconstruct biological signals. We chose DCA [20] to further recover biological noise of mRNA expression. DCA considers a read count matrix as input and uses a deep-learning-based autoencoder to infer parameters of count distribution. The output of DCA is a DCA denoised count matrix with the same shape as the input count matrix. As with RPKM, we normalized DCA output with effective gene length and library size to create DCA normalized counts, and then calculated RCV (Figure 1c,d and Figure A1c,d,k,l). We deemed both the RCV RPKM and the RCV DCA normalized count as expression noises of each mRNA in downstream analysis. The DCA denoised count matrix measures the total noise, including biological noise and technical noise. The input count matrix measures reconstructed biological noise, but may also include bias caused by the DCA algorithm.

**Figure A2:**
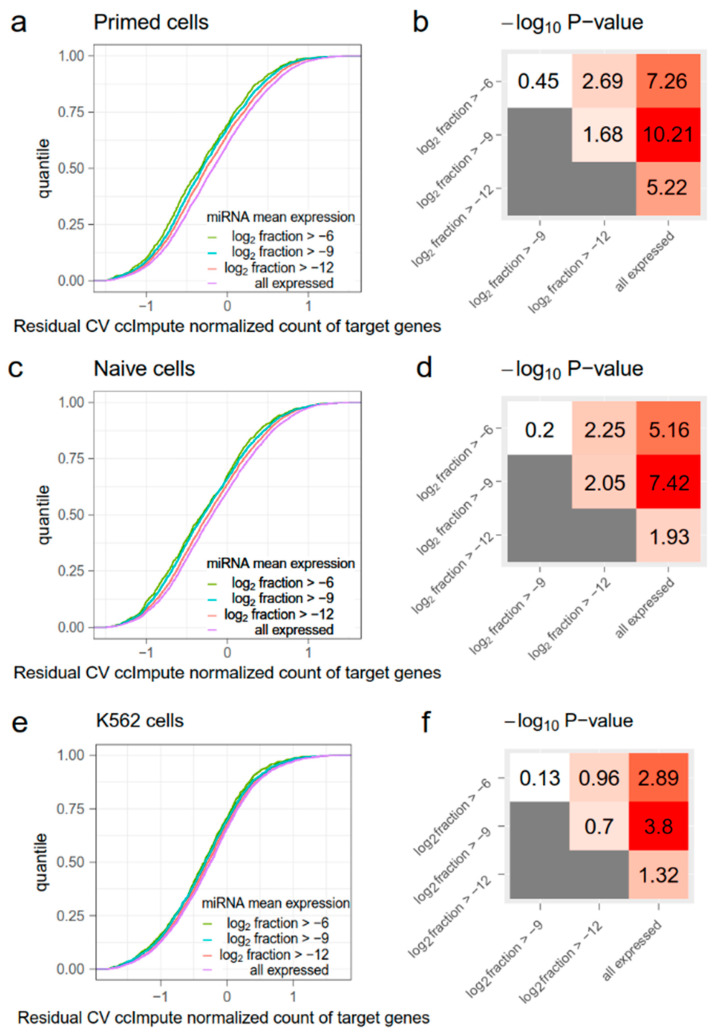
The evaluation of the noise-reduction effect of miRNAs using ccImpute. The organization of this figure is the same as for Figure 1. (**a**,**c**,**e**) Cumulative distribution functions (CDFs) of residual coefficient of variation (RCV) normalized counts of target genes in primed cells (**a**), naïve cells (**c**), and K562 cells (**e**). Target genes are grouped by miRNA mean expression levels. As the miRNA mean expression level increases, the RCV ccImpute normalized count of their target genes significantly decreases. (**b**,**d**,**f**) Kolmogorov–Smirnov (KS)-test −log10 (*p*) values between CDFs in (**a**,**c**,**e**). Red indicates higher statistical significance and white indicates lower statistical significance.

**Figure A3:**
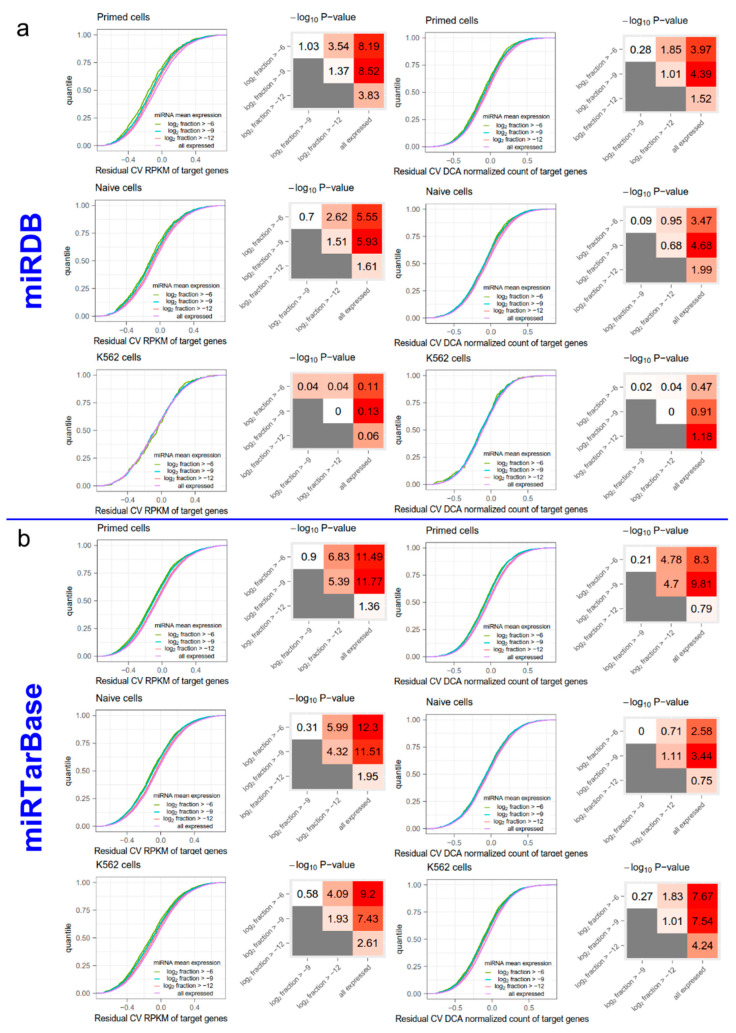
The evaluation of the noise-reduction effect of miRNAs using two distinct miRNA target prediction databases miRDB (**a**) and miRTarBase (**b**). The organization of this figure is the same as for Figure 1. Please refer to the legend of Figure 1 for details.

The effect of miRNA on target gene expression noise may vary according to expression level. Therefore, we grouped miRNAs according to their expression levels (the mean fraction in all miRNAs). Four groups were partitioned based on a gradient of the mean fraction in the logarithmic scale (Figure 1e, Table A1). We first predicted the target mRNAs of miRNAs in each group, using TargetScan [22]. We then compared the expression noise (RCV RPKM and RCV DCA normalized count) of target mRNAs between every two groups. We found that the target mRNAs of miRNAs with higher expression level generally had lower RCV RPKMs (Figure 1e and Figure A1e,m). The KS test results showed significant differences between the several pairs of the distributions of RCV RPKMs (Figure 1f and Figure A1f,n). Notably, the statistical significance increased as the difference in miRNA expression levels increased. These results suggest that the total noise of predicted target mRNAs is anti-correlated with the expression level of miRNAs. Given that the mRNAs with higher technical noise are not enriched in the predicted targets of specific miRNAs, the results show that miRNAs reduce the expression noise of target mRNAs. For RCV DCA normalized counts, we observed a similar pattern; specifically, highly expressed miRNAs strongly reduced the expression noise of target mRNAs (Figure 1g,h and Figure A1g,h,o,p). This suggests that the noise-reduction effect of miRNAs can be observed in recovered biological noises. To validate this finding, we repeated the analysis using a different noise-reduction method (ccImpute [21], Figure A2) and two additional miRNA target prediction databases (miRDB [23] and miRTarBase [24], Figure A3). All the results were similar and support the conclusion that miRNAs reduce the expression noise of target mRNAs.

**Figure 2:**
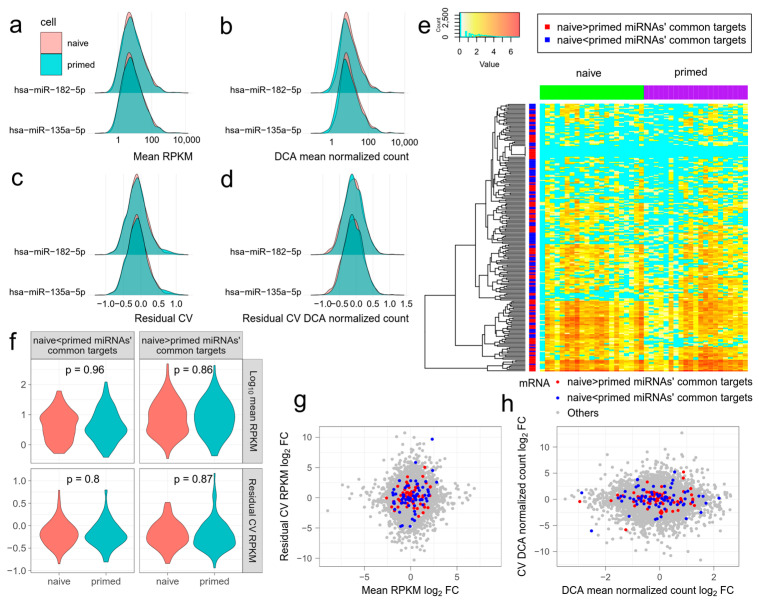
The effect of differentially expressed miRNAs on mean expression level and the noise of target mRNAs. miRNA and mRNA data are from GSE81287 naive and primed embryonic stem cells (ESCs). (**a–d**) Distributions of mean expression level ((**a**) mean RPKM, (**b**) mean DCA normalized count) and noise of mRNAs ((**c**) residual coefficient of variation (RCV) RPKM, (**d**) RCV DCA normalized count) targeted by differentially expressed miRNAs. hsa-miR-182-5p and hsa-miR-135a-5p are representative differentially expressed miRNAs, with higher expression in naive and primed ESCs, respectively. The distributions are almost identical in naive and primed ESCs. (**e**) Log(#count + 1) of the commonest mRNAs targeted by differentially expressed miRNAs in naive and primed ESCs. MRNAs targeted by miRNAs with higher expression in naive and primed ESCs have similar patterns. Zero expression is shown in cyan. (**f**) Violin plots showing the mean expression levels and noises of the commonest mRNAs targeted by differentially expressed miRNAs in naive and primed ESCs. (**g**,**h**) The log fold change of the mean expression level and noise of all the mRNAs in naive and primed ESCs. The commonest mRNAs targeted by differentially expressed miRNAs do not generally deviate from the origin. (**g**) Mean RPKM and RCV RPKM. (**h**) Mean DCA normalized count and RCV DCA normalized count.

We next examined the effect of differentially expressed miRNAs in a number of cell types. The miRNA expression levels of naive ESCs and primed ESCs were analyzed in GSE81287. Faridani et al. identified 327 differentially expressed miRNAs (adjusted *p* < 0.05) [8], of which 159 showed higher expression in naive ESCs and 168 showed higher expression in primed ESCs. We used their results to avoid inconsistencies with the primary analysis. For each differentially expressed miRNA, we predicted its target mRNAs, and compared their mean expression and noise. We found that no miRNA showed a significant difference in either its log mean expression or the noise of target mRNAs (*t*-test, Benjamini FDR > 0.05). In fact, the distributions of mean expression and noise were nearly identical for each miRNA (Figure 2a–d). This suggests that the effect of a single miRNA on expression noise is too weak to be detected.

Combinatorial regulation of miRNAs has been shown to cause substantial reduction in target gene expression and noise [5]. Therefore, we next compared the combinatorial effect of all differentially expressed miRNAs, between naive ESCs and primed ESCs. We separated all the differentially expressed miRNAs into two groups. One group contained miRNAs with higher expression in naive ESCs and the other contained miRNAs with higher expression in primed ESCs. For each mRNA, we counted the frequency that it is targeted by two groups of miRNAs. Then, we selected 100 mRNAs with the highest frequency in each group, namely, the most common targets. The 100 mRNAs in the two groups had similar expression patterns and did not agglomerate with mRNAs from the same group in hierarchical clustering (Figure 2e). Neither the expression level nor noise of 100 mRNAs differed significantly between naive ESCs and primed ESCs in each group (Figure 2f, *t*-test, *p* > 0.05 for mean RPKM and RCV). The log fold change of mean expression or noise of some mRNAs was greatly increased or reduced. However, none of the mRNAs of the naive ESCs or primed ESCs showed an overall inclination to increase or reduce mean expression and noise (Figure 2g,h). These results further show that using the applied methodologies, the effect of miRNA regulation on expression noise of target mRNAs is hard to detect in single cells.

## 4. Discussion

In this study, we showed that miRNAs slightly reduce the expression noise of target genes; however, this effect is easily masked by strong technical noise from scRNA-seq. As we delineate below, prior to running scRNA-seq, improvements can be implemented in experimental design and computational analysis, aimed to reduce technical noise. Such guidelines may be useful for experiments that evaluate the effect of miRNAs on mRNA expression from scRNA-seq.

The means by which miRNAs regulate target mRNA were addressed in several studies. scRNA-seq now enables observing the effect of this regulation at a higher resolution. Here, we accessed two available datasets (GSE81287, GSE114071) that provide both single-cell mRNA and single-cell miRNA profiling, to analyze miRNA regulation on the expression noise of target mRNAs. We endeavored to properly measure the expression noise of mRNAs by accounting for the effect of mean expression level, and used a denoising algorithm, DCA, to further reconstruct biological noise. We compared the expression noise of target mRNAs, between miRNAs with different expression levels in a group of cells, and between differentially expressed miRNAs in a number of cell types. Although some results indicated that the total noise of predicted target mRNAs is anti-correlated with the expression level of miRNAs, other results revealed that miRNA regulation is generally mild and its noise reduction effect on target mRNAs is not easily discerned.

To date, very few studies have combined single-cell mRNA and single-cell miRNA profiling. In our experiments, we attempted to identify the technical noise that is part of the total noise, in order to estimate true biological variability. However, the datasets we used were not generated with this intention; hence, a large proportion of the technical noise could not be separated from the biological noise. Several methods have been developed to quantify technical noise and biological noise from scRNA-seq data. According to these methods, bulk-cell mixture controls or external spike-ins are required in the decomposition of total noise [12,26,27]. However, neither of these techniques were used in GSE81287 and GSE114071; this precluded completely separating technical noise and biological noise. Moreover, amplification is also a major source of noise in scRNA-seq. UMIs are widely considered the best approach to reduce amplification noise, but both of the datasets we used implemented the read-based Smart-seq2 protocol rather than UMI-based protocols. As spike-ins and UMIs were not available in the datasets, we chose DCA to estimate biological noise because it does not require spike-ins, and is applicable to both read-based and UMI-based expression data. However, the denoised data output by DCA and other methods may be limited by the algorithms, and are not capable of recovering true biological noise. If future studies were to include spike-ins and UMIs, biological noise may be better identified.

## 5. Conclusions

We propose several guidelines for future studies on the effect of miRNAs on gene expression with scRNA-seq. (i) The use of a UMI-based protocol rather than a read-based protocol in mRNA sequencing will greatly reduce amplification noise [13]. (ii) The use of spike-ins in mRNA sequencing and noise decomposition tools will increase the potential of decomposing total noise into technical noise and biological noise with spike-ins [12,27]. Such tools may facilitate examining the effect of miRNA on true biological noise. (iii) The sequencing of sufficient samples and sequencing of each library to a nearly saturated depth such as one million reads [28] will increase the accuracy of estimations of mean expression level and noise. (iv) Multiple miRNA target prediction databases (TargetScan, miRDB, miRTarBase, etc.) should be considered in the interpretation of experimentally validated data. The results from one database can be misleading and could introduce additional technical noise. Therefore, validation by different methods is needed. We expect that future studies with improved experimental design will better address the problems.

## Data Availability

All data used in this study are available in GEO database, including GSE81287 and GSE114071.

## Appendix A

**Table A1:** The numbers of miRNAs and target genes in GSE81287 and GSE114071. Target genes of multiple miRNAs are the union of target genes of each miRNA.

	GSE81287 Primed		GSE81287 Naive		GSE114071	
MiRNA Mean Expression	MiRNA Number	Target Gene Number	MiRNA Number	Target Gene Number	MiRNA Number	Target Gene Number
log2 fraction > −6	9	3258	13	3898	5	1443
log2 fraction > −9	43	8046	71	7102	24	3274
log2 fraction > −12	149	10812	178	10562	55	6736
All expressed	519	12381	509	12402	262	8849
